# Alternative diagnoses in neonates undergoing therapeutic hypothermia for presumed hypoxic–ischemic encephalopathy

**DOI:** 10.3389/fped.2026.1879783

**Published:** 2026-08-05

**Authors:** Tugrul Donmez, Ozge Serce Pehlevan, Ayla Gunlemez

**Affiliations:** 1 European Vocational College, Kocaeli Health and Technology University, Kocaeli, Türkiye; 2 Division of Neonatology, Department of Pediatrics, Kocaeli University, Körfez, Türkiye

**Keywords:** differential diagnosis, hypoxic–ischemic encephalopathy, neonatal encephalopathy, non-hypoxic–ischemic etiologies, perinatal asphyxia, therapeutic hypothermia

## Abstract

**Background:**

Early identification of hypoxic–ischemic encephalopathy (HIE) remains challenging during the first hours of life, and therapeutic hypothermia (TH) is often initiated before the underlying diagnosis can be established.

**Objective:**

The primary aim of this work is to compare the clinical characteristics, laboratory findings, and short-term outcomes of neonates treated with TH for presumed HIE according to their predominant underlying diagnosis.

**Methods:**

We retrospectively reviewed all neonates who received TH for suspected HIE between 2019 and 2023. Infants were categorized according to the predominant underlying diagnosis identified following comprehensive clinical, neurophysiological, neuroimaging, metabolic, genetic, and subspecialty evaluations. Demographic, clinical, neurologic, and laboratory parameters—including inflammation- and oxidative stress-related indices—were compared between groups.

**Results:**

Among 92 neonates treated with TH, nine (10%) were subsequently found to have non-hypoxic–ischemic etiologies of neonatal encephalopathy. Baseline neurological severity assessed by Sarnat staging and initial amplitude-integrated electroencephalography patterns did not differ between groups. However, infants in the non-HIE group demonstrated significantly higher rates of myocardial injury (77% vs. 22%, *p* < 0.001) and severe thrombocytopenia (66% vs. 25%, *p* = 0.016). These infants also had substantially longer hospitalizations [median (25P–75P): 28 (17–38) vs. 7 (5–11) days, *p* = 0.002] and higher mortality rates (55.6% vs. 6%, *p* = 0.001). The median day of death was earlier in the non-HIE group [median (25P–75P): 3 (1–57) vs. 16 (8–77) days; *p* = 0.30]. However, it was not statistically significant. Inflammatory and oxidative stress indices did not differ significantly between groups.

**Conclusion:**

Approximately 10% of neonates undergoing therapeutic hypothermia for presumed HIE were ultimately found to have non-hypoxic–ischemic causes of neonatal encephalopathy despite similar early neurologic presentations. These infants experienced greater systemic morbidity and poorer short-term outcomes. While the small and heterogeneous non-HIE cohort warrants cautious interpretation, disproportionately severe systemic manifestations—particularly myocardial injury and thrombocytopenia—may represent early clinical clues prompting broader diagnostic evaluation beyond hypoxic–ischemic injury.

## Introduction

Neonatal encephalopathy (NE) is a clinical syndrome characterized by disturbed neurological function in the newborn (1). Although NE is frequently used interchangeably with hypoxic–ischemic encephalopathy (HIE), the two terms are not synonymous. HIE refers specifically to brain injury resulting from perinatal hypoxia–ischemia, whereas NE is a descriptive syndrome encompassing a broad spectrum of underlying etiologies, including metabolic, infectious, genetic, structural, vascular, and neuromuscular disorders (1–3).

Because many clinical, biochemical, and neurophysiological features overlap among these conditions, distinguishing HIE from alternative causes of NE during the first hours after birth remains challenging. Metabolic acidosis, low Apgar scores, seizures, abnormal amplitude-integrated electroencephalographic (aEEG) findings, and the need for extensive resuscitation may occur in both hypoxic–ischemic and non-hypoxic conditions, making early differentiation of the underlying cause challenging (1, 3, 4). Although NE affects approximately 2–8 per 1,000 live births worldwide, only a subset of affected infants ultimately fulfills diagnostic criteria for HIE, underscoring the importance of accurate etiologic evaluation (2).

Therapeutic hypothermia (TH) is the standard neuroprotective treatment for moderate-to-severe HIE in high-income settings and has been shown to reduce mortality and long-term neurodevelopmental disability when initiated within the first 6 h after birth (5). Because this therapeutic window is narrow, clinicians are frequently required to initiate treatment before the underlying diagnosis can be established. Consequently, TH is often commenced in infants with presumed HIE while investigations for metabolic, genetic, structural, infectious, or other causes of NE remain ongoing. This strategy prioritizes timely neuroprotection in infants with true hypoxic–ischemic injury, recognizing that the underlying diagnosis often becomes apparent only after comprehensive diagnostic evaluation (3, 6). Recent commentaries have emphasized that the term NE should be regarded as an initial clinical diagnosis rather than a final etiologic diagnosis. Failure to distinguish HIE from other causes of NE may obscure appropriate diagnosis, delay disease-specific management, and complicate both clinical decision-making and interpretation of research findings (3, 6). Nevertheless, further characterization of infants who undergo therapeutic hypothermia for presumed HIE but are subsequently found to have non-hypoxic–ischemic causes of NE is needed.

Accordingly, the primary aim of this study was to compare the clinical characteristics, laboratory findings, neurophysiological features, and short-term outcomes of infants who underwent TH for presumed HIE and were subsequently classified as having either HIE-consistent NE or NE due to non-hypoxic–ischemic etiologies. As a secondary objective, we evaluated whether selected inflammation- and oxidative stress-related composite indices—including the systemic immune-inflammation index (SII), hemoglobin–albumin–lymphocyte–platelet (HALP) score, uric acid-to-albumin ratio (UA/Alb), and lactate dehydrogenase-to-albumin ratio (LDH/Alb)—could facilitate early etiologic differentiation, as these biomarkers have shown potential associations with hypoxic–ischemic injury but have not been adequately evaluated for distinguishing HIE from non-hypoxic–ischemic causes of NE (7–11).

## Methods

This retrospective cohort study was conducted at a single tertiary neonatal intensive care unit (NICU) serving as a regional referral center for TH. The study period encompassed 4 years from 1 January 2019 to 1 January 2023. After approval by the institutional ethics committee (27 May 2024/E.594842), data were extracted from electronic medical records.

### Patient selection and grouping

Eligible infants (≥36 weeks' gestation and >2,000-g) were admitted to the NICU within the first 12 h after birth. As our institution serves as a regional tertiary referral center, some infants were transferred from outside hospitals after birth. In selected infants admitted between 6 and 12 h of age, TH was initiated following individualized consultant assessment, with careful consideration of the potential risks and benefits, particularly when the diagnosis of moderate-to-severe HIE remained clinically plausible despite delayed presentation. TH was initiated primarily in infants who met institutional diagnostic criteria for moderate-to-severe encephalopathy based on clinical examination using the modified Sarnat and Sarnat scales and/or abnormal aEEG findings. In addition, because neurological findings in NE may evolve during the first hours after birth, TH was not initiated solely on the basis of a single Sarnat examination. In our unit, infants with initially mild (Stage 1) encephalopathy were considered for TH if neurological abnormalities progressed on serial examinations, seizures developed, and/or aEEG demonstrated abnormalities consistent with moderate-to-severe encephalopathy. In these situations, the decision to initiate TH was made on an individual basis by the attending neonatal consultant after careful assessment of the overall clinical picture and the potential risks and benefits of treatment. Exclusion criteria included major congenital anomalies and known or suspected chromosomal disorders at the time of the TH decision.

A total of 92 infants met inclusion criteria and were retrospectively categorized into two groups:

(a) HIE-consistent NE (NE with predominant hypoxic–ischemic etiology) (*n* = 83).
(b) Non-HIE group (predominant underlying diagnosis other than HIE) (*n* = 9). The predominant underlying diagnosis was established through review of all available clinical, neurophysiological, neuroimaging, metabolic, genetic, cardiologic, and subspecialty consultation data. Diagnostic investigations varied according to the suspected underlying condition and were not identical for all patients. Because alternative diagnoses and hypoxic–ischemic injury are not necessarily mutually exclusive, infants were categorized according to the predominant underlying diagnosis identified during the diagnostic workup rather than the complete exclusion of hypoxic–ischemic injury.

TH was applied in accordance with the national guideline for NE (12). TH was initiated in infants with arterial pH ≤7.00 or base excess ≥16 mmol/L when these findings were accompanied by moderate-to-severe encephalopathy on the modified Sarnat and Sarnat examination and/or abnormal aEEG patterns. For borderline cases with pH between 7.00 and 7.15 or base deficit between 12 and 16 mmol/L, TH was commenced if at least two additional clinical findings (e.g., low Apgar score, prolonged resuscitation, or seizures) were present or if moderate-to-severe encephalopathy and/or an abnormal aEEG patterns were identified. In infants who did not fully meet the biochemical criteria but in whom HIE remained clinically suspected, the decision to initiate TH was made on an individual basis by the attending neonatal consultant after careful assessment of the overall clinical presentation, neurological findings, aEEG findings, and the potential risks and benefits of treatment. The target core temperature was maintained between 33.0°C and 34.0°C for 72 h, followed by gradual rewarming at a rate of 0.5°C per hour.

Background activity on aEEG was classified according to the Hellström–Westas voltage classification as normal (continuous normal voltage), moderately abnormal (discontinuous normal voltage or burst suppression), or severely abnormal (low voltage or flat trace) (13).

Cranial ultrasonography was performed as part of the initial evaluation before initiation of TH and repeated during or after treatment when clinically indicated to identify alternative causes of NE, such as intracranial hemorrhage or arterial ischemic stroke. The institutional neonatal MRI protocol included conventional brain MRI sequences (T1- and T2-weighted imaging) together with diffusion-weighted imaging (DWI) and apparent diffusion coefficient (ADC) maps. MRI imaging was routinely performed at approximately postnatal day 5, provided the infant was clinically stable. Brain MRI examinations were interpreted by an experienced pediatric neuroradiologist as part of routine clinical care. Neuroimaging findings were reviewed retrospectively from radiology reports and clinical records. A standardized MRI injury scoring system (e.g., Barkovich, Weeke, or modified Rutherford score) was not routinely applied, as the primary aim of the present study was etiologic classification rather than quantitative grading of hypoxic–ischemic brain injury; nevertheless, complete MRI sequence parameters and imaging findings were systematically recorded for all examinations. MRI findings contributed to establishing the underlying diagnosis but were interpreted together with the complete clinical, neurophysiological, metabolic, genetic, and multidisciplinary evaluation rather than serving as the sole determinant of diagnosis.

### Data collection

Clinical and laboratory data were retrieved from electronic hospital records. Demographic variables (gestational age, birth weight, sex, delivery mode), perinatal indicators (Apgar scores, time to TH initiation), and initial laboratory results obtained at hospital admission—before initiation of TH whenever applicable, including arterial pH, base excess, lactate, troponin, serum creatinine, and complete blood count parameters—were recorded. Neurologic assessment included Modified Sarnat staging and aEEG background patterns obtained during the first 24 h. Clinical outcomes occurring during hospitalization including severe thrombocytopenia (platelet count <100,000/mm^3^) and acute kidney injury defined as a serum creatinine >1.5 mg/dL or urine output <1 mL/kg/h for ≥24 h were also systematically recorded.

### Inflammation and oxidative stress indices

(a) To evaluate whether hematologic and biochemical markers of inflammation and oxidative stress might contribute to early etiologic differentiation, several composite indices were calculated using laboratory values obtained before initiation of therapeutic hypothermia (7–11): SII = (platelet count × neutrophil count)/lymphocyte count.
(b) HALP score (hemoglobin–albumin–lymphocyte–platelet) = (hemoglobin × albumin  ×  lymphocyte count)/platelet count.
(c) Uric acid-to-albumin ratio (UA/Alb) = serum uric acid (mg/dL)/serum albumin (g/dL).
(d) Lactate dehydrogenase-to-albumin ratio (LDH/Alb) = serum LDH (U/L)/serum albumin (g/dL).

### Statistical analysis

Statistical analyses were performed using IBM SPSS Statistics for Windows, version 29.0 (IBM Corp., Armonk, NY, USA). Continuous variables were assessed for normality using the Shapiro–Wilk test. Non-normally distributed data were reported as medians and percentiles, and compared using the Mann–Whitney *U*-test. Categorical variables were presented as frequencies and percentages. Given the small sample size of the non-HIE group and unequal group distributions, categorical comparisons were conducted using Fisher's exact test.

Because of the exploratory nature of the study and the small size of the non-HIE cohort, formal adjustment for multiple comparisons was not performed, and findings should be interpreted as hypothesis-generating. For Mann–Whitney *U* comparisons, effect size was calculated as *r* = *Z*/√*N*, where *Z* is the standardized test statistic and *N* is the total number of observations. Effect sizes were interpreted as small (*r* = 0.10), medium (*r* = 0.30), and large (*r* ≥ 0.50). A two-tailed *p*-value <0.05 was considered statistically significant for descriptive purposes. Time to death was analyzed using Kaplan–Meier survival curves, and differences between the HIE-consistent NE and non-HIE groups were compared using the log-rank (Mantel–Cox) test.

## Results

A total of 92 neonates underwent TH during the study period. Of these, 83 (90%) were classified as having HIE-consistent NE, whereas 9 (10%) were classified as having non-HIE encephalopathy following completion of the diagnostic evaluation. The non-HIE group comprised two neuroblastomas, two chromosomal/syndromic disorders, two inherited metabolic disorders, one congenital neuromuscular disorder, one skeletal dysplasia, and one complex congenital disorder with central hypoventilation. Detailed clinical, neurophysiological, neuroimaging, and diagnostic characteristics of these nine infants are summarized in Table 1. Among infants in the non-HIE group, neuroimaging findings varied according to the underlying diagnosis.

**Table 1:** Characteristics of infants in the non-HIE group who underwent therapeutic hypothermia.

Case	Underlying diagnosis	Perinatal findings leading to TH	Initial biochemical findings^a^	Sarnat stage	aEEG/EEG findings	Neuroimaging findings	Key diagnostic clue	Primary diagnostic modality
1	Osteogenesis imperfecta with multiple fractures and intracranial hemorrhage	Apgar 0/1, 20 min cardiopulmonary resuscitation	pH 6.90, BE −20 mmol/L, lactate 14 mmol/L, troponin-T 2.5 ng/mL, platelets 35 × 10^9^/L	Stage 3	Inactive/flat trace	Extensive extra-axial hemorrhage, diffuse cerebral edema, multiple skull fractures	Multiple skull and long-bone fractures, severe intracranial hemorrhage, and bilateral hemothorax inconsistent with isolated HIE	Skeletal survey and neuroimaging
2	Congenital myotonic dystrophy	Fetal bradycardia, Apgar 3/4	pH 7.14, BE −12.9 mmol/L, lactate 10 mmol/L, troponin-T 0.18 ng/mL	Stage 2	Moderately depressed background	Normal cranial ultrasonography; MRI unavailable	Maternal and sibling history of myotonic dystrophy with profound neonatal hypotonia	Family history and clinical genetic evaluation
3	Syndromic disorder with central hypoventilation and limb anomalies	Apgar 6/7, respiratory failure	pH 6.9, BE −15 mmol/L, lactate 7 mmol/L	Stage 3	Right-sided suppression	MRI without diffusion restriction; susceptibility signal abnormalities in basal ganglia and brainstem	Central hypoventilation, recurrent apnea, and digital agenesis	Clinical phenotype and neuroimaging
4	Down syndrome with congenital heart disease	Apgar 5/6, need for resuscitation	pH 7.08, lactate 7.4 mmol/L, platelets 104 × 10^9^/L	Stage 2	Moderately depressed background	Mega cisterna magna	Dysmorphic features and congenital cardiac anomalies	Genetic evaluation and echocardiography
5	Pyruvate metabolism disorder with intestinal malrotation	Apgar 5/8, deterioration within 1 h after birth	pH 6.80, BE −22 mmol/L, lactate 20 mmol/L, troponin-T 0.85 ng/mL	Stage 3	Moderately depressed background	Hydrocephalus and intraventricular hemorrhage	Persistent severe lactic acidosis and refractory metabolic derangements	Metabolic investigations
6	Pyridoxine-dependent epilepsy	Meconium aspiration, Apgar 4/6	pH 7.08, BE −14 mmol/L	Stage 2	Initial suppression with normalization after pyridoxine administration	Normal brain MRI	Marked electroclinical improvement following pyridoxine administration	Pyridoxine challenge test and metabolic/genetic evaluation
7	Trisomy 13 with tetralogy of Fallot and omphalocele	Home delivery, Apgar 3/5	pH 6.90, BE −18 mmol/L, lactate 13 mmol/L	Stage 2	Moderately depressed background	Cranial ultrasonography showed grade I hemorrhage; MRI unavailable	Multiple congenital anomalies including omphalocele and congenital heart disease	Genetic testing and cardiac evaluation
8	Adrenal neuroblastoma	Meconium-stained delivery, Apgar 2/5	pH 7.01, BE −14.7 mmol/L, lactate 12 mmol/L	Stage 1	Moderately suppressed background	Normal cranial ultrasonography MRI unavailable	Prenatal and postnatal detection of adrenal mass	Abdominal imaging and oncology evaluation
9	Mediastinal neuroblastoma	Apgar 2/5, 5 min cardiopulmonary resuscitation	pH 6.90, BE −18 mmol/L, lactate 12 mmol/L	Stage 1	Moderately suppressed background	Normal cranial ultrasonography MRI unavailable	Mediastinal mass causing tracheal deviation and respiratory compromise	Thoracic imaging and oncology evaluation

BE, base excess.

a Laboratory values obtained before initiation of therapeutic hypothermia.

In the overall cohort, initial aEEG patterns were classified as normal in 12 infants (12.9%), moderately abnormal in 69 (74%), and severely abnormal in 11 (11.8%). According to modified Sarnat staging, 46 infants (49%) presented with Stage 1, 35 (37.6%) with Stage 2, and 11 (11.8%) with Stage 3 encephalopathy.

Gestational age and birth weight were similar between groups: 38 weeks (25P–75P; 37–40) vs. 38 weeks (25P–75P; 37–38), *p* = 0.27; 3,300 g (25P–75P; 2,780–3,620) vs. 3,200 g (25P–75P; 2,920–3,575), *p* = 0.99. Cesarean delivery was significantly less frequent among infants with HIE-consistent NE [34 (41%)] compared with the non-HIE group [8 (88%); *p* = 0.011]. Apgar scores at 5 min, cord blood gas parameters, Sarnat staging, and initial aEEG patterns showed no significant differences between groups (Table 2). In terms of systemic complications, myocardial injury (elevated troponin) occurred in 77% of infants in the non-HIE group vs. 22% in the HIE group (*p* < 0.001). Severe thrombocytopenia (<100,000/mm^3^) was also more common in the non-HIE group (66% vs. 25%; *p* = 0.016). Infants in the NE group with alternative etiologies had a significantly longer NICU stay [28 days (25P–75P; 17–38) vs. 7 days (25P–75P; 5–11); *p* = 0.002] and higher mortality (55.6% vs. 6%; *p* = 0.001). Although deaths occurred earlier among infants in the non-HIE group (median day 3 vs. 16), this difference did not reach statistical significance (log-rank *p* = 0.30). Other outcome parameters are summarized in Table 3. Evaluation of inflammatory and oxidative stress indices (SII, UA/Alb, LDH/Alb ratio, HALP score) showed no significant differences between the groups. Effect sizes for all comparisons were small (*r* < 0.20) (Table 4).

**Table 2:** Comparison of infants with and without hypoxic–ischemic encephalopathy among those receiving therapeutic hypothermia (TH).

Variable	Hypoxic–ischemic encephalopathy (*N* = 83)	Alternative etiologies of NE (*N* = 9)	*p*-Value
Gestational age (weeks), median (25P–75P)	38 (37–40)	38 (37–38)	0.27
Birth weight (g), median (25P–75P)	3,300 (2,780–3,620)	3,200 (2,920–3,575)	0.99
Male sex, *n* (%)	55 (66)	5 (55)	0.71
Cesarean section, *n* (%)	34 (41)	8 (88)	0.011
1-min Apgar score, median (25P–75P)	4 (2–5)	3 (2–5)	0.84
5-min Apgar score, median (25P–75P)	6 (5–7)	5 (4–7)	0.64
Resuscitation, *n* (%)			
Positive pressure ventilation	17 (20)	1 (11)	0.89
Intubation	48 (58)	6 (67)	
Adrenaline	18 (21)	2 (22)	
Cord pH, median (25P–75P)	6.9 (6.8–7.05)	6.9 (6.8–7.06)	0.89
Cord bas excess, median (25P–75P)	−17(−19 to −13)	−16.5(−18 to −9)	0.69
Lactate level, median (25P–75P)	12 (8–17)	12 (8–13)	0.79
Sarnat staging, *n* (%)			
Stage 1	43 (51)	3 (33)	0.10
Stage 2	32 (39)	3 (33)	
Stage 3	8 (9.6)	3 (33)	
Initial aEEG pattern, *n* (%)			
Normal	12 (14)	0	0.071
Moderately abnormal	63 (76)	6 (66)	
Severely abnormal	8 (9)	3 (33)	
Convulsion, *n* (%)	23 (27)	5 (55)	0.12
Time to TH initiation (h), median (25P–75P)	4 (3–5)	6 (3–8)	0.19

**Table 3:** Short-term clinical outcomes.

Variable	Hypoxic–ischemic encephalopathy	Alternative etiologies of NE	*p*-Value
	(*N* = 83)	(*N* = 9)	
Need for inotropic support, *n* (%)	69 (83)	9 (100)	0.34
Renal failure, *n* (%)	46 (55)	4 (44)	0.52
Hepatic failure, *n* (%)	75 (90)	7 (77)	0.28
Pulmonary hypertension, *n* (%)	9 (10)	0 (0.0)	0.59
Troponin elevation/myocardial injury, *n* (%)	19 (22)	7 (77)	*<0.001*
International normalized ratio elevation, *n* (%)	64 (77)	5 (55)	0.22
Platelet<100,000/mm^3^, *n* (%)	21 (25)	6 (66)	*0*.*016*
Respiratory support, *n* (%)			
Free-flow oxygen	3 (3)	0	0.99
Nasal ventilation	14 (16)	1 (11)	
Invasive ventilation	67 (80)	8 (88)	
Length of hospitalization (days), median (25P–75P)	7 (5–11)	28 (17–38)	*0*.*002*
Mortality, *n* (%)	5 (6)	5 (55.6)	*0*.*001*
Mortality day, median (25P–75P)	16 (8–77)	3 (1–57)	*0*.*30*

Italic values indicate statistical significance (*p* < 0.05).

**Table 4:** Comparison of systemic inflammatory and oxidative stress indices according to etiology of neonatal encephalopathy (NE).

Variable	Hypoxic–ischemic encephalopathy	Alternative etiologies of NE	*p*-Value	(Effect size)
	(*N* = 83)	(*N* = 9)		
SII, median (25P–75P)	626 (320.6–1,042.8)	476 (243.9–715.4)	0.30	0.10
Uric acid-to-albumin Ratio, median (25P–75P)	2 (1.70–2.59)	2.3 (1.93–2.85)	0.47	0.07
LDH to albumin ratio, median (25P–75P)	363 (252.7–598.0)	396 (267.4–667.0)	0.76	0.03
HALP score, median (25P–75P)	1.14 (0.76–1.75)	1.5 (0.81–3.73)	0.44	0.08

SII, systemic immune-inflammation index; was calculated as (platelet × neutrophil)/lymphocyte. HALP score was calculated as (hemoglobin × albumin × lymphocyte count)/platelet count.

## Discussion

Our study demonstrates that approximately 1 in 10 infants who underwent TH for presumed HIE was ultimately found to have non-HIE encephalopathy. Importantly, initial neurological severity—assessed by Sarnat staging, aEEG, and biochemical markers of perinatal acidosis—was comparable between groups, underscoring the substantial diagnostic overlap between HIE and non-hypoxic–ischemic causes of NE during the early postnatal period. Despite similar neurological presentations, infants in the non-HIE group experienced significantly higher mortality and greater systemic morbidity than those with HIE-consistent NE. Collectively, these findings highlight the diagnostic uncertainty encountered during the first hours after birth, when treatment decisions must be made within a narrow neuroprotective window before the results of comprehensive diagnostic investigations become available.

Our findings support the evolving concept that NE should be regarded as an initial clinical syndrome rather than a definitive diagnosis. As emphasized by Branagan et al., a diagnosis of NE should prompt immediate evaluation and consideration of TH when HIE is suspected, while the underlying cause should be clarified through ongoing diagnostic investigations (14). This concept is particularly relevant because genetic, metabolic, structural, and other non-asphyxial disorders may closely mimic HIE during the first hours after birth, precisely when therapeutic decisions must be made (14–16).

This diagnostic uncertainty was well illustrated in our cohort. Infants in the non-HIE group frequently underwent TH before the underlying diagnosis was established, with several diagnoses becoming apparent only after metabolic investigations, genetic testing, neuroimaging, or multidisciplinary evaluation. Most infants were referred from outside hospitals, where incomplete obstetric and perinatal documentation further complicated the initial diagnostic evaluation. As our institution serves as a regional tertiary referral center for critically ill outborn neonates, referral-related delays likely contributed to the interval between birth and establishment of the underlying diagnosis. In addition, several underlying disorders were associated with prenatal abnormalities or suggestive family histories. However, these findings were often non-specific or insufficient to establish the diagnosis before birth. Definitive genetic diagnoses were also not immediately available because confirmatory testing required referral to external laboratories, with turnaround times extending beyond the narrow therapeutic window for initiating TH. Consequently, TH was initiated whenever HIE remained a clinically plausible diagnosis while definitive diagnostic investigations continued.

Importantly, our findings should not be interpreted as suggesting that therapeutic hypothermia (TH) should be delayed until an underlying diagnosis has been established. Although unnecessary TH may expose some infants without HIE to treatment-related adverse effects (e.g., invasive monitoring, sedation, coagulopathy, or cardiovascular instability), the potential harm associated with withholding TH from an infant with true HIE generally outweighs the risks of treating a limited number of infants who are subsequently found to have non-hypoxic–ischemic causes of NE. As TH remains the only established neuroprotective therapy for moderate-to-severe HIE and must be initiated within the first 6 h after birth to achieve maximal benefit (17, 18), treatment decisions inevitably precede definitive neuroimaging, metabolic investigations, and genetic testing. Consequently, initiation of TH before the underlying diagnosis is fully established is an inherent feature of current neonatal practice. Rather than supporting more restrictive eligibility criteria, our findings highlight the need for more accurate early diagnostic tools capable of improving etiologic discrimination without delaying initiation of TH.

Because a diagnosis of HIE carries important therapeutic, prognostic, and medicolegal implications, clinicians may reasonably favor initiating TH when the diagnosis is clinically plausible but not yet definitive (19). In this context, our findings emphasize that atypical clinical evolution, disproportionate extracerebral organ involvement, or a clinical course that deviates from the expected trajectory of HIE should prompt early reassessment of the underlying diagnosis and a broader diagnostic evaluation rather than continued attribution to hypoxic–ischemic injury alone.

Although neurological severity at presentation was comparable between groups, infants in the non-HIE group exhibited a distinctly different systemic clinical profile. They had significantly higher troponin concentrations, more frequent severe thrombocytopenia, greater mortality, and longer hospital stays than infants with HIE-consistent NE. Similar observations have been reported in previous studies demonstrating poorer outcomes among infants with non-HIE NE (14, 20). These findings likely reflect the heterogeneous mechanisms underlying the diagnoses. Genetic syndromes, inherited metabolic disorders, and multisystem diseases may cause myocardial dysfunction or hematologic abnormalities through mechanisms distinct from perinatal hypoxic–ischemic injury. Therefore, these abnormalities should not be regarded as disease-specific biomarkers but rather as clinical clues suggesting that the underlying disease process extends beyond isolated hypoxic–ischemic brain injury and warrants comprehensive metabolic, genetic, cardiac, and neuroimaging assessment.

An important implication of our findings is that the identification of a non-HIE underlying diagnosis does not necessarily exclude concomitant hypoxic–ischemic injury. NE is frequently multifactorial, and genetic, metabolic, or structural disorders may coexist with—or increase susceptibility to—perinatal hypoxic–ischemic injury (14). Infants in our cohort were classified according to the predominant underlying diagnosis identified after comprehensive clinical evaluation including neurophysiological findings, neuroimaging when available, metabolic investigations, genetic testing, cardiologic assessment, and multidisciplinary consultation. Brain MRI contributed to establishing the underlying diagnosis when available but was interpreted together with the complete clinical and diagnostic evaluation rather than as the sole determinant of diagnosis. Because MRI could not be obtained in all infants owing to early death or clinical instability, concomitant hypoxic–ischemic injury cannot be completely excluded in every individual case. We also exploratorily evaluated several inflammation- and oxidative stress-related composite biomarkers as potential adjuncts for early etiologic differentiation. None of the investigated indices, including SII, HALP score, UA/Alb ratio, and LDH/Alb ratio, differed significantly between groups, despite prior reports of their elevation in HIE, and all effect sizes were small (7–11). One possible explanation is that some infants in the non-HIE group may also have experienced concomitant hypoxic–ischemic injury, resulting in overlapping inflammatory and oxidative stress responses between groups. In addition, severe neonatal illness itself, regardless of the underlying diagnosis, may trigger similar systemic inflammatory and oxidative stress pathways, further reducing the discriminatory capacity of these biomarkers. Consequently, these findings should be considered exploratory and hypothesis-generating rather than evidence against their biological relevance. Future prospective multicenter studies incorporating standardized neuroimaging, comprehensive diagnostic investigations, and larger numbers of infants in the non-HIE group are required to determine whether these biomarkers have a role in early etiologic stratification.

An additional observation was the significantly higher frequency of cesarean delivery among infants in the non-HIE group compared with those with HIE-consistent NE. One possible explanation is that underlying genetic, structural, neuromuscular, or metabolic disorders may present antenatally with reduced fetal movements, congenital anomalies, or non-reassuring fetal status, prompting obstetric intervention before labor progression. Conversely, a proportion of hypoxic–ischemic injuries arise from acute intrapartum events occurring during labor, which may account for the relatively higher proportion of vaginal deliveries in the HIE group. However, because of the limited number of infants in the non-HIE group and the retrospective design, this observation should be interpreted cautiously and requires confirmation in larger prospective studies.

This study has several limitations. First, the non-HIE cohort was small and clinically heterogeneous, limiting statistical power and precluding disease-specific analyses. Second, the retrospective single-center design resulted in non-standardized diagnostic evaluations, and brain MRI was unavailable in a subset of infants because of early death or clinical instability. Third, the biomarker analyses were exploratory and no adjustment for multiple comparisons was performed. Fourth, brain MRI was performed at a fixed institutional time point (approximately day 5 of life) using conventional T1- and T2-weighted sequences together with DWI/ADC mapping. Although DWI is highly sensitive to acute hypoxic–ischemic injury during this early window, conventional sequence abnormalities continue to evolve over the following days, and delayed imaging performed around days 9–10 of life may more accurately delineate the final extent of irreversible injury (21, 22). Early DWI findings may also be atypical or falsely reassuring in neonates with underlying genetic or metabolic disorders mimicking HIE who nonetheless undergo TH (23), a consideration particularly relevant to the non-HIE group in our cohort. Serial or delayed MRI, where feasible, may therefore provide additional diagnostic information beyond a single early scan (21). Finally, although all examinations were interpreted by an experienced pediatric neuroradiologist as part of routine clinical care, a standardized quantitative MRI scoring system was not applied, consistent with recent calls for greater standardization of MRI timing and injury classification in neonates with NE/HIE (24, 25). Therefore, these findings should be considered hypothesis-generating and require confirmation in larger prospective multicenter studies. Despite these limitations, our study provides one of the larger single-center descriptions of infants in the non-HIE group identified after initiation of therapeutic hypothermia and reflects real-world clinical decision-making in a regional referral center.

In conclusion, NE remains a heterogeneous clinical syndrome in which distinguishing HIE from non-hypoxic–ischemic causes of NE during the therapeutic window is often challenging. Although TH should not be delayed in infants who meet established treatment criteria, clinicians should remain alert to atypical or severe clinical features and pursue early, systematic diagnostic evaluation when appropriate. Improving the timely recognition of conditions that mimic HIE may facilitate disease-specific management, enhance prognostic accuracy, and optimize the use of TH.

## Data Availability

The raw data supporting the conclusions of this article will be made available by the authors, without undue reservation.

## References

[1] Russ JB Simmons R Glass HC. Neonatal encephalopathy: beyond hypoxic-ischemic encephalopathy. Neoreviews. (2021) 22:e148–62. 10.1542/neo.22-3-e148 33649088

[2] Kurinczuk JJ White-Koning M Badawi N. Epidemiology of neonatal encephalopathy and hypoxic-ischaemic encephalopathy. Early Hum Dev. (2010) 86(6):329–38. 10.1016/j.earlhumdev.2010.05.010 20554402

[3] Sandoval Karamian AG Mercimek-Andrews S Mohammad K Molloy EJ Chang T Chau V. Neonatal encephalopathy: etiologies other than hypoxic-ischemic encephalopathy. Semin Fetal Neonatal Med. (2021) 26(5):101272. 10.1016/j.siny.2021.101272 34417137

[4] O'Dea M Sweetman D Bonifacio SL El-Dib M Austin T Molloy EJ. Management of multi organ dysfunction in neonatal encephalopathy. Front Pediatr. (2020) 8:239. 10.3389/fped.2020.00239 32500050 PMC7243796

[5] Fink A Berg MT Soll RF Fiander M Hunter K Aagerup J. Therapeutic hypothermia for newborns with hypoxic-ischaemic encephalopathy. Cochrane Database Syst Rev. (2026) 4(4):CD016345. 10.1002/14651858.CD016345 41954139 PMC13063367

[6] Gunn AJ Soul JS Vesoulis ZA Ferriero DM. The importance of not increasing confusion around neonatal encephalopathy and hypoxic-ischemic encephalopathy. Pediatr Res. (2024) 95:871–2. 10.1038/s41390-023-03001-6 38158415

[7] Ceran B Alyamaç Dizdar E Beşer E Karaçağlar NB Sarı FN. Diagnostic role of systemic inflammatory indices in infants with moderate-to-severe hypoxic ischemic encephalopathy. Am J Perinatol. (2024) 41(3):248–54. 10.1055/a-1673-1616 34666380

[8] Toptan HH Tezel KG Tezel O Ataç Ö Vardar G Gülcan Kersin S. Inflammatory and hematologic liver and platelet (HALP) scores in hypothermia-treated hypoxic-ischemic encephalopathy (HIE. Children (Basel). (2024) 11(1):72. 10.3390/children11010072 38255385 PMC10814453

[9] Mohamed RA Ali IA. Role of neutrophil/lymphocyte ratio, uric acid/albumin ratio and uric acid/creatinine ratio as predictors to severity of preeclampsia. BMC Pregnancy Childbirth. (2023) 23(1):763. 10.1186/s12884-023-06083-6 37904105 PMC10614385

[10] Jeon SY Ryu S Oh SK Park JS You YH Jeong WJ. Lactate dehydrogenase to albumin ratio as a prognostic factor for patients with severe infection requiring intensive care. Medicine (Baltimore). (2021) 100(41):e27538. 10.1097/MD.0000000000027538 34731152 PMC8519202

[11] Elsadek AE FathyBarseem N Suliman HA Elshorbagy HH Kamal NM Talaat IM. Hepatic injury in neonates with perinatal asphyxia. Glob Pediatr Health. (2021) 8:2333794X20987781. 10.1177/2333794X20987781 33614837 PMC7868451

[12] Akisu M Kumral A Canpolat FE. Turkish Neonatal Society Guideline on neonatal encephalopathy. Turk Pediatri Ars. (2018) 53(Suppl 1):32–44. 10.5152/TurkPediatriArs.2018.01805 PMC6568287 31236017

[13] Chock VY Rao A Van Meurs KP. Optimal neuromonitoring techniques in neonates with hypoxic ischemic encephalopathy. Front. Pediatr. (2023) 11:1138062. 10.3389/fped.2023.1138062 36969281 PMC10030520

[14] Branagan A Molloy EJ Badawi N Nelson KB. Causes and terminology in neonatal encephalopathy: what is in a name? Neonatal encephalopathy, hypoxic-ischemic encephalopathy or perinatal asphyxia. Clin Perinatol. (2024) 51(3):521–34. 10.1016/j.clp.2024.04.015 39095093

[15] Woodward KE Murthy P Mineyko A Mohammad K Esser MJ. Identifying genetic susceptibility in neonates with hypoxic-ischemic encephalopathy: a retrospective case series. J Child Neurol. (2023) 38(1–2):16–24. 10.1177/08830738221147805 36628482

[16] Lenahan A Mietzsch U Wood TR Callahan KP Weiss EM Miller DE. Characteristics, genetic testing, and diagnoses of infants with neonatal encephalopathy not due to hypoxic ischemic encephalopathy: a cohort study. J Pediatr. (2023) 260:113533. 10.1016/j.jpeds.2023.113533 37269901

[17] Zanelli SA Wusthoff CJ Lucke AM Kaufman DA Eichenwald E Ambalavanan N. Therapeutic hypothermia for neonatal hypoxic-ischemic encephalopathy: clinical report. Pediatrics. (2026) 157(2):e2025073627. 10.1542/peds.2025-073627 41581784

[18] Proietti J Boylan GB Walsh BH. Regional variability in therapeutic hypothermia eligibility criteria for neonatal hypoxic-ischemic encephalopathy. Pediatr Res. (2024) 96:1153–61. 10.1038/s41390-024-03184-6 38649726 PMC11521984

[19] Bhorat I Buchmann E Soma-Pillay PS Nicolaou E Pistorius L Smuts I. Cerebral palsy and its medicolegal implications in low-resource settings—the need to establish causality and revise criteria to implicate intrapartum hypoxia: a narrative review. S Afr Med J. (2023) 113(7):29–34. 10.7196/SAMJ.2023.v113i6.229 37882043

[20] Nelson KB Bingham P Edwards EM Horbar JD Kenny MJ Inder T. Antecedents of neonatal encephalopathy in the Vermont Oxford Network Encephalopathy Registry. Pediatrics. (2012) 130(5):878–86. 10.1542/peds.2012-0714 23071210 PMC4074646

[21] Sotelo E, Sharon D, Gagoski B, Ellen Grant P, Singh E, Inder TE. Insights from serial magnetic resonance imaging in neonatal encephalopathy in term infants. *Pediatr Res.* (2024) 95:1–9. 10.1038/s41390-024-03258-5 38907045

[22] Garvey AA El-Shibiny H Yang E Inder TE El-Dib M. Differences between early and late MRI in infants with neonatal encephalopathy following therapeutic hypothermia. Pediatr Res. (2023) 94(3):1011–7. 10.1038/s41390-023-02580-8 37024670

[23] Misser SK Archary M. Mimickers of hypoxic-ischaemic brain injury in term neonates: what the radiologist should know. S Afr J Radiol. (2024) 28(1):a2810. 10.4102/sajr.v28i1.2810 PMC11019187 38628264

[24] Mohammad K Reddy Gurram Venkata SK Wintermark P Farooqui M Beltempo M Hicks M. Consensus approach for standardization of the timing of brain magnetic resonance imaging and classification of brain injury in neonates with neonatal encephalopathy/hypoxic-ischemic encephalopathy: a Canadian perspective. Pediatr Neurol. (2025) 166:16–31. 10.1016/j.pediatrneurol.2025.01.021 40048833

[25] Di Stasi M Plantulli A Varsalone FF Locatelli G Paolella C Frauenfelder G. Neuroimaging of neonatal brain post therapeutic hypothermia: a practical guide to the non-pediatric neuroradiologist. Front Radiol. (2026) 6:1722473. 10.3389/fradi.2026.1722473 41782739 PMC12953461

